# The impact of opioid agonist treatment on fatal and non-fatal drug overdose among people with a history of opioid dependence in NSW, Australia, 2001-2018: findings from the OATS retrospective linkage study.

**DOI:** 10.23889/ijpds.v7i3.1787

**Published:** 2022-08-25

**Authors:** Nicola Jones, Louisa Degenhardt, Matthew Hickman, Suzanne Nielsen, Sarah Larney, Timothy Dobbins, Robert Ali

**Affiliations:** 1 National Drug and Alcohol Research Centre; 2 University of Bristol; 3 Monash University; 4 Université de Montréal; 5 Centre de Recherche du Centre Hospitalier de l'Université de Montréal; 6 University of New South Wales; 7 The University of Adelaide

## Objectives

There are critical periods of mortality risk at onset and cessation of opioid agonist treatment. We aim to determine whether non-fatal overdose followed the same pattern as fatal overdose, comparing the first 4 weeks of treatment and treatment cessation and the remainder time off treatment, with the remainder treatment time, to determine intervention markers.

## Approach

Retrospective cohort study of people with a history of opioid agonist treatment using linked New South Wales data. The incidence of non-fatal overdose hospitalization; emergency department presentation; and fatal overdose from national death records were compared. Rates were calculated using generalized estimating equations adjusting for demographics, year, and recent health and incarceration events.

## Results

The rate of an emergency department drug overdose presentation was highest. It was more than three-fold the rate of opioid non-fatal overdose hospitalisation and 14 times higher than fatal opioid overdose. It was also twice the rate of non-opioid non-fatal overdose hospitalisation.

Fatal overdose was lowest while in treatment. This differed from the measures of non-fatal overdose, the overdose rate was elevated in the first four weeks in treatment as well as the first four weeks post treatment.

## Conclusion

Retention on opioid agonist treatment is protective against drug related overdose. There is elevated risk of non-fatal overdose at treatment initiation that is not evident for fatal overdose, however the first month of treatment cessation is a critical period for both non-fatal and fatal overdose. These findings emphasize the importance of treatment retention and interventions for polysubstance overdose at cessation.

